# Scotch Doctors and the Voluntary System

**Published:** 1920-12-04

**Authors:** 


### THE FUTURE OF HOSPITALS.

### Scotch Doctors and the Voluntary System.

"The Future of the Hospitals under the Ministry of Health" was discussed at the recent annual meeting of the Medical Guild at Edinburgh.

Dr. A. Blackhall-Morison, of London, declared that the future of the hospitals has become a political question, and that since the politician appeared the medical profession has suffered. There are now 12,000 medical men opposed to the Ministry of Health, and these compare favourably with the lambs who first followed Dr. Addison's Bill into the promised pasture of State Medicine.

Dr. Ebenezer Duncan, of Glasgow, contrasted the German system of State hospitals and the American system of graduated patients with the British or voluntary system. With us, he said, the special hospitals in particular are abused, and the surgical wards of the general hospitals. Payments from patients able to contribute should be extended. The Ministry of Health is an autocracy, for which reason their Medical Guild opposed it utterly in medical matters. His connection with the Bellahouston Hospital under the Ministry of Pensions had shown the greatest difficulty of obtaining proper treatment for the patients, because of a system of indenting for all surgical and medical appliances. They had to indent, a month before for everything they wanted, and perhaps to wait for six months before they received it. In emergencies they had resorted to telegrams and letters, which evoked an impertinent letter from some subordinate official in Edinburgh, insisting on indentation a month in advance.

Emeritus Professor Caird said that even in his time the class of patient in the Royal Infirmary has changed, and now numbers many in the eye and ear wards who should never be there. Confiscation of the hospital means bureaucratic government for the medical profession, which would sink to the level of a set of lower-grade Civil Service clerks.

Dr. V. T. Greenyer, of the National Medical Union, said that if the Government appropriate the voluntary hospitals the better part of the profession will be infected with the apathy and want of enterprise that already permeates the panel doctors.

Dr. Alexander James, of Edinburgh, said that, notwithstanding the increase of population, the subscriptions to the Royal Infirmary have not been proportionately maintained. The reason is that Christian charity has been choked by so-called "State charity," of which there has been a boom during the past fifty years. He sketched the growing mass of legislation in Education, Public Health, Old-age Pensions, Health Insurance, and the institutions created thereby. He hoped the proposal to make the voluntary hospitals rate-paid hospitals would be set aside at once. It would be throwing good money after bad. He was opposed also to pay wards in the infirmary. The need for more money can be met by giving charity a fresh chance, and reconsidering the evil effects of State charity legislation.

Professor Robinson saw no help for it but an annual subsidy, but such subsidy must not involve bureaucratic control by Government or municipal bodies.

Dr. James Ritchie, of Edinburgh, said that their experience of Government control did not warrant them to agree to the nationalisation of hospitals.

Emeritus Professor Russell said that the difficulties of the voluntary hospitals have whetted the appetite of the bureaucrat, who desires either to beat them out or to dominate them.

It was finally resolved that before any change is made in the voluntary system the Medical Guild is of opinion that immediate steps be taken to devise a scheme for patients' contributions towards the cost of their maintenance.

